# Diabetes Screening Through Community Pharmacies in England: A Cost-Effectiveness Study

**DOI:** 10.3390/pharmacy7010030

**Published:** 2019-03-22

**Authors:** David Wright, Richard Little, David Turner, Tracey Thornley

**Affiliations:** 1 School of Pharmacy, University of East Anglia, Norwich NR4 7TJ, UK; 2 Health Economics Consultancy, Cambridge CB23 7EQ, UK; Richard@little4.demon.co.uk; 3 Norwich Medical School, University of East Anglia, Norwich NR4 7TJ, UK; David.A.Turner@uea.ac.uk; 4 Boots UK Ltd, 1 Thane Road, Beeston, Nottingham NG90 1BS, UK; Tracey.thornley@boots.co.uk; 5 University of Nottingham, University Park, Nottingham NG7 2RD, UK

**Keywords:** community pharmacy, screening, type II diabetes, cost-effectiveness

## Abstract

Community pharmacies are commonly used to screen for patients with diabetes. The aim of this paper is to estimate the cost per test and cost per appropriately referred patient from a pharmacy perspective using a one-year decision tree model. One-way sensitivity analysis was performed to estimate the effect of geographical location and patient self-referral rate. Data was used from 164 patients screened and located in an area with average social deprivation and largely white European inhabitants and 172 patients in an area with higher social deprivation (lower than average ability to access society’s resources) and a mixed ethnicity population in England. The diabetes screening consisted of initial risk assessment via questionnaire followed by HbA1c test for those identified as high risk. The cost per person screened was estimated as £28.65. The cost per appropriately referred patient with type 2 diabetes was estimated to range from £7638 to £11,297 in deprived mixed ethnicity and non-deprived areas respectively. This increased to £12,730 and £18,828, respectively, if only 60% of patients referred chose to inform their general practitioner (GP). The cost per test and identification rates through community pharmacies was similar to that reported through medical practices. Locating services in areas of suspected greater diabetes prevalence and increasing the proportion of patients who follow pharmacist advice to attend their medical practice improves cost-effectiveness.

## 1. Introduction

With over three million people in the UK diagnosed with diabetes in 2014, it was estimated that there were another 590,000 undiagnosed [1]. Uncontrolled type 2 diabetes is related to an increased risk of developing cardiovascular disease, likelihood of lower limb amputation, kidney disease, diabetes retinopathy and dementia [1], and in 2011 was estimated to cost the National Health System (NHS) £8.8bn and society £13.9bn. By 2035/36, diabetes is expected to change from accounting for 10% of total NHS resource to 17% [2], with one third of this being due to complications [2].

Diabetes is diagnosed either by identification of elevated blood glucose tests (fasting > 7.0 mmol/L and random > 11.1 mmol/L) or HbA1c > 48 mmol/mol (6.5%) [3]. Impaired glucose tolerance (IGT) or pre-diabetes refers to those individuals whose blood glucose tests are elevated, but not sufficiently high enough to warrant a diagnosis of diabetes [4]. Pre-diabetes is more accurately identified through fasting blood glucose when compared to use of an HbA1c result alone, and consequently those with HbA1C levels indicative of pre-diabetes are referred to as being at ‘high risk of diabetes’ [5]. Pharmacological and lifestyle interventions are beneficial in reducing the risk of progression to type 2 diabetes mellitus in people with impaired fasting glucose or IGT [6].

Early identification and treatment of diabetes is known to reduce the incidence of complications [7], and therefore is a public health strategy which could significantly contribute to reducing this predicted increase in resource utilisation [8]. Screening activity within a population increases the prevalence of diagnosed diabetes [9], and results in cases being identified 3.3 years earlier on average [10].

Researchers in the USA and Germany have demonstrated that diabetes screening is likely to be cost-effective if focused on the under 75 population [11] and those who are overweight [12]. Screening for diabetes in patients between 45 and 75 with intervention for those identified as being at high risk of diabetes in the UK is believed to be cost-effective [8]. The UK the government recommends that diabetes screening should be focused on patients from known high risk ethnic groups and with increased body mass index [4].

Strategies to improve cost-effectiveness have included focusing screening on those at greater risk, e.g. people in specific ethnic groups and people with greater body mass indices [4], using screening methods with greater sensitivity and specificity and utilizing different approaches to participant identification and inclusion.

One such approach is the use of diabetes risk assessment tools [13]. Whilst these are less effective than standard blood tests at identifying patients with diabetes [14], they are used to reduce the number of unnecessary tests [15]. In such cases, the point at which a blood test is recommended to be undertaken has to be carefully considered. A low cut off point results in a large number of unnecessary tests and resultant high cost per case detected. Conversely, high cut off points increases the chances of cases being missed and large numbers of screenings required to obtain a small number of positive results [16], It is important that the likelihood of false positives is limited, as such unnecessary referrals are known to cause patient harm [17].

Researchers in the UK and Netherlands who tested different approaches to recruit patients for screening found that patients were more willing to undertake screening if they were approached whilst accessing healthcare for a different purpose [18]. Patients and the general population access community pharmacy services on a regular basis, therefore providing an opportunity to screen patients. Whilst it is assumed that the cost of screening through community pharmacies is likely to be similar to other routes, the actual cost of per appropriately referred patient is unknown [19].

Opportunistic screening by a community pharmacist within Thailand, without risk assessment, resulted in 2 patients being diagnosed following 397 patients being screened [20]. Whilst a greater number of patients were identified as at risk, a significant proportion of patients referred to their general practitioner (GP) chose not to attend a screening [20]. and this has been seen in a similar study [21]. Krass et al. showed that risk assessment followed by blood tests in Australian community pharmacies resulted in fewer referrals and greater uptake by patients than when using risk assessment only, i.e., 76% chose to visit their GP when both tests were undertaken, compared to 63% with just the questionnaire [13]. Patients who don’t self-refer could be positive and thereby delaying diagnosis and treatment. The effect of different referral uptakes on cost-effectiveness is also unknown.

## 2. Aim

The aim of this paper is to determine the cost per patient with type 2 diabetes appropriately referred to their GP through community pharmacies using the current UK model within different geographical settings and assuming different rates of referral to GPs.

## 3. Methods

### 3.1. Ethics Approval

As an economic modelling study based on anonymized service data, and as a service evaluation, ethical approval was not sought for this study.

### 3.2. Patient and Public Involvement

There was no patient and public involvement in the design of the study or preparation of this paper.

### 3.3. Service Data

To inform the model, data were collected from the diabetes screening services provided in Leicester and Surrey for a six-month period in 2014. The Leicester area has a higher than average level of social deprivation and a larger proportion of the population is drawn from ethnic minorities. The Surrey area has lower than average social deprivation and a relatively homogenous white European population.

The pharmacy-based screening service, which was used to provide data regarding service delivery to underpin the model, was aimed at individuals over the age of 18 and unlikely to be pregnant and is summarized below:

- Completion of standardised type 2 diabetes screening customer service record form (CRF), consisting of a valid and reliable tool for diabetes screening [13] and questions required by the UK government for screening purposes, by the individual with support from a healthcare assistant (HCA) to estimate their risk level for diabetes.
- Signposting to further services as appropriate, e.g., weight-management, smoking cessation for individuals identified at low risk (score less than 16).
- Offering an HbA1c non-fasting finger prick blood test by the pharmacist for individuals determined to be at moderate or high risk of diabetes (score greater than 15)
- Providing a follow up pharmacist appointment in three months to individuals identified as being at high risk of diabetes via the HbA1C test (between 6.1% (43mmol/mol) and 6.4% (47mmol/mol)).
- Advising individuals identified as being likely to have type 2 diabetes via the HbA1C test (≥6.5% (48mmol/mol)) to see their GP for further testing and appropriate treatment.
- Communication of all HbA1c test results to the individual’s GP with their consent.

Data available from this service to inform model creation was that which was sent to the company head office and consisted of:

- Pharmacy location
- Age group, gender and ethnicity of patient
- Risk assessment score derived from the customer report form [22]
- HbA1c result

### 3.4. Effectiveness Modelling

The service described above was a pragmatic community pharmacy-led service and for this reason we had no data on the true prevalence of type 2 diabetes (T2D) in these samples, or on the proportion who would have been advised to consult their GP, who actually had diabetes. Furthermore, we had no data on what would have happened to the patients in the absence of this service.

**Figure 1:**
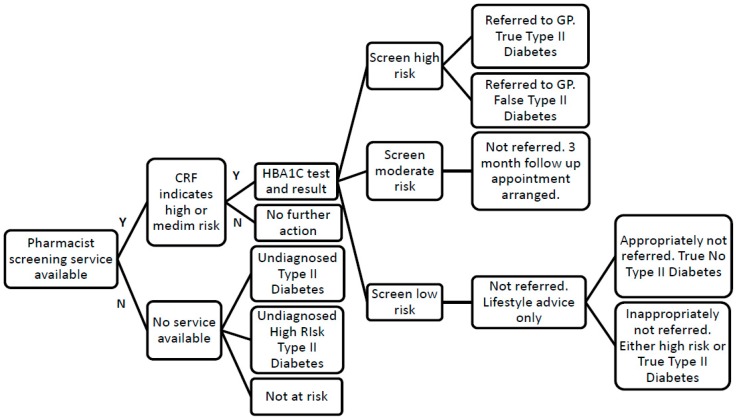
Service flow diagram used to underpin economic decision tree.

Spreadsheet-based models were constructed to estimate these values using the data from the screening services, supplemented with data from the literature, as per Figure 1. We had service level data from Leicester and Surrey on the number of patients suspected of have diabetes identified and referred. It was unclear how many of these were true positives. The prevalence data used to generate estimates of actual numbers of individuals with diabetes and pre-diabetes are given in Table 1. For Leicester, the proportions expected to have T2D, and those at high risk of diabetes, were obtained from the ADDITION study [23]. This source was used for both the white and ethnic minority population. For Surrey, the prevalence of T2D in both white and ethnic minority individuals were taken from a published report [1]. The proportion of high risk individuals for Surrey was taken from a modelling study [5]. As we had no specific data on the proportion of individuals at high risk of diabetes for Surrey, this was assumed to be the same as for ethnic minorities (Leicester) [23].

For T2D, we assumed that individuals who had T2D would not access the service (as the service is designed for identification purposes only), so the prevalence of T2D was multiplied by the proportion who would have undiagnosed T2D (15%) [1]. The performance of the CRF and HbA1c testing was based on these estimates of prevalence multiplied by estimates of the sensitivity and specificity from the literature. The sensitivity (0.81) and specificity (0.45) of the CRF questionnaire was taken from Gray et al [13]. Values from Tankover et al. were used for the non-fasting HbA1c blood test [22]. These were: type 2 diabetes—sensitivity 0.86, specificity 0.92, and pre-diabetes—sensitivity 0.71, specificity 0.64. These values were used to estimate the number of true positives from CRF screenings, and the expected numbers of cases of T2D who were declared positive (true positives) by the HbA1c test, and hence would be appropriately referred to their GP. This case, appropriately referred, was our measure of effectiveness in this analysis.

### 3.5. Comparator

Standard care would be no pharmacy-led screening and hence no individuals with suspected diabetes would be referred from pharmacies. Therefore, it was assumed that the cases identified would be additional to those without screening.

### 3.6. Costs of Pharmacy Led Screening and Cost-Effectiveness

Data was collected from the pilot studies to inform the expected costs of providing this service in pharmacies. Costs were identified through observation of the service by a health economist (RL), measured via feedback from service providers, valued through the use of national databases or provision of costs from the service provider and calculated from the pharmacy perspective.

It was assumed that the CRF would be completed by a healthcare assistant (HCA) (non-regulated member of the pharmacy team, commonly also referred to as pharmacy assistant) and that this was estimated to require 15 minutes. A cost per hour of £12.88 was used for HCA time [24]. If the CRF indicated moderate or high risk of diabetes, an HbA1c test would be performed by a pharmacist.

The HbA1c analyser and initial training costs were annualised over 5 years (it being unrealistic to assume that they would only be used for one year) and with a 3.5% annuitisation rate [25]. Additional pharmacist time was required to carry out the test and discuss results with service users. Again, it was assumed that 15 minutes of time would be required and a cost per hour of pharmacist time in providing direct patient contact of £73 was used [26]. Other consumables required to carry out the test were recorded from the pilot evaluation. It was assumed that there was no cost associated with offering a screening to patients who subsequently declined participation. It was assumed that all individuals who were screened and identified at high risk were referred to their GP for confirmatory diagnosis.

The average number of individuals seen within each pharmacy was calculated to inform service costs over a one-year period. Those pharmacies who saw less than 5 patients in six months were deemed not to be active participants and not included in the calculation. As the time frame was less than 1 year, discounting was not used. All costs cited in the paper are expressed in 2017 prices, using the Curtis, Lesley A. and Burns, Amanda (2017) Unit Costs of Health and Social Care 2017 [26]. In order to evaluate the service, we used the above data on costs and numbers of referrals to estimate the cost per diabetes case referred (cost per true positive, i.e., patients who actually have the disease).

### 3.7. Sensitivity Analysis

In the base case analysis we assumed that 100% of individuals identified as having HBA1c ≥ 6.5 would actually visit their GP. However, in reality, less than this may do so. A one-way sensitivity analysis was performed to determine how estimates of cost-effectiveness change depending on the proportion referred and who actually attended, ranging from 60% (worst case scenario based on Krass et al. [14]) to 100% (best case scenario).

## 4. Results

Data were provided from six pharmacies in Leicester (n = 172) and five from Surrey (n = 164). Table 2 summarises patient demographics and test results. The CRF test indicated medium to high risk (>16) for 64 (37%) and 40 (24%) patients in Leicester and Surrey, respectively. A number of HBA1c results for moderate to high risk individuals were unavailable and therefore estimates for the results for all individuals scored as moderate or high risk by the CRF are provided.

Table 3 provides the results of the modelling exercise, where results are predicted based on prevalence and screening performance. These were generated by multiplying the numbers in the different areas by probabilities obtained from the literature. White inhabitants of Leicester are provided as an illustrated example. Of the 97 individuals screened by means of the CRF questionnaire, 53.9 (55.6%) were predicted to screen high to moderate risk. From these, it was estimated that 0.6% have diabetes (true positive for T2D), 2.5% have pre-diabetes (true positive for pre-diabetes) and 96.9% were false positives. All 53.9 individuals identified as high/moderate risk were assumed to have HbA1c tests and 16.1 to screen positive in an HbA1c test for T2D and high risk of diabetes. There were 4.46 individuals who screened as high risk for T2D, which represents 27.8% of all those who tested positive on the HbA1c test. Out of these, 0.29 individuals have T2D and 4.18 individuals are false positives. All 4.46 individuals would be referred to their GP for evaluation, with 0.29 representing the effectiveness measure, i.e., those with true type 2 diabetes.

Table 4 provides a summary of the unit costs for service delivery based on locating the service within Leicester. To calculate the average size of pharmacies providing screening, we divided the total number of patients by the number of pharmacies. Two pharmacies in Leicester provided data on less than five patients (1 & 4 patients) and were excluded. As it was not possible to identify the pharmacy providing screenings for 3 patients, these patients were also excluded. Consequently, data from these 8 screenings were excluded, leaving 328 patients included in the analysis. After excluding the two pharmacies with less than 5 patients, there were 9 pharmacies deemed to be actively engaged. This total of 328 patients over 9 pharmacies gave a mean of 36.5 patients per pharmacy in the six-month study period, equating to 73 patients per year (95% CI 46 to 98).

The assumptions underpinning these cost estimates are also given in Table 4. Costs are divided into those associated with providing CRF screening and those associated with follow-up HbA1c testing. Costs associated with CRF screening include training, marketing, printing, and HCA time and equate to £6.44 per patient. Table 4 provides an estimate of 40.9 patients out of 73 who screened medium or high risk. This was used as the denominator for estimates of the unit cost of providing HbA1c. The total estimate of cost per test for HbA1c in this setting was £40. However, since only 41 patients received HbA1c testing per pharmacy, the average cost per person of the combined screening program was £29.

Table 5 presents the average cost per appropriately referred individual. These were generated from information presented in Table 3 and Table 4. Results are presented for Leicester and Surrey, in both cases data from white inhabitants’ and ethnic minority inhabitants’ screenings are combined. We present costs of CRF screening and HbA1c tests. Table 5 presents the total number appropriately referred individuals, i.e., those who have T2D and were referred by the pharmacy to their GP practice. Table 5 also presents the total cost of screening, which is the total numbers of individuals screened in each area multiplied by the cost per person screened taken from Table 4. The additional cost of screening is divided by the number of appropriately referred individuals to give an estimate of cost-effectiveness in terms of cost per appropriately referred individual. The cost-effectiveness of screening is given as £7638 and £11,297 per appropriately referred patient for Leicester and Surrey, respectively. This increases to £12,730 and £18,828 if only 60% of patients who are recommended to attend their GP actually do so.

## 5. Discussion

The estimated cost per test as delivered within the underpinning service was only marginally greater than that reported in other medical practice-based studies [8]. The data provided from the two locations suggested that the proportion of patients identified as at high risk of diabetes was also similar to that seen within a Leicester medical practice-based population screen [27]. Consequently, we can assume that a community pharmacy based service is likely to provide similar results to that provided from a medical practice.

The cost per appropriately referred individual from a community pharmacy ranges from approximately £7638 to £18,828, being dependent on geographical location and ability to motivate patients to go to their GP for confirmatory testing. This can be compared with a medical practice-based service targeting only high risk patients, with no uptake data, and the cost per case of type 2 diabetes identified was estimated to be between £471 and £1689 [28].

The cost per person with true diabetes referred was approximately one third lower in Leicester compared to Surrey. This was due to the higher prevalence of diabetes in Leicester compared to Surrey. Consequently, additional diabetes screenings through pharmacies may be better put to use being targeted to those populations where need is greatest.

Evidence suggests that proactive screening of individuals at high risk is likely to identify diabetes just over 3 years earlier than where screening is not provided [10]. The question for primary care service commissioners is whether a total cost of £7638 to refer one true case of diabetes earlier than would otherwise be the case is justifiable, as well as the question of at what point the cost becomes prohibitive. If £18,828 is seen as representing good value, then the service should be provided nationally. However, in a resource-constrained NHS, it is important that services provide good value and this can be achieved by ensuring that people identified as being at high risk of diabetes within a community pharmacy do make and attend follow up appointments with their GP to undertake confirmatory tests. Further research is required to determine actual adherence to referral recommendations and to identify how to enhance this in order to improve service efficiency.

It is known that patients with pre-diabetes and diabetes are more likely to be prescribed lipid-lowering and hypertensive treatments [29] and one further approach to enhancing service efficiency in community pharmacies may be to use this prescribing information to further focus their screening strategy.

A larger number of patients with pre-diabetes were however identified within both services and this provides an opportunity to intervene and reverse pre-diabetes, with this approach believed to be likely to be cost-effective [8]. Providing a service through healthy living pharmacies which mirrors the NHS diabetes prevention program [30], and includes an intervention, maybe worthy of testing.

## 6. Strengths and Limitations

The analysis is based on data from real pharmacy services located in two geographically distinct areas, national statistics and reported sensitivity and specificity data for the different elements and is therefore relatively robust. Whilst the greater proportion of females and white Europeans identified as accessing the service in both areas may not represent patients within high risk groups, it does reflect the patient population who access community pharmacy services and therefore the cost-effectiveness analysis is based upon a representative dataset. That there was a small amount of screening results data available, however, provides some imprecision.

The study relied on time estimates for staff costs, and sensitivity analysis surrounding this could be undertaken, however, the greatest effect is the sensitivity/specificity of the tests and prevalence estimates. Estimates are based on the data sent to the head office, which is a small sub-set of individuals actually screened, and potential bias in reporting cannot be discounted. Whilst it would have been preferable to take a whole health system perspective when estimating costs, development of such a model was beyond the project’s remit. A wider perspective would have implied wider costs, but this doesn’t invalidate the perspective adopted.

## 7. Conclusions

Community pharmacy-based diabetes risk assessment and screening services were found to have a similar cost per test and ability to detect people at risk of diabetes to that provided from medical practices. Locating services in areas of greatest prevalence of unidentified diabetes and ensuring that patients identified as at risk self-refer to their medical practice improves cost-effectiveness.

## Figures and Tables

**Table 1:** Proportion of population estimated to have different conditions.

Demographic	Proportion		
	Normal Glucose Tolerance ^#^	High Risk of Diabetes	Type 2 Diabetes
White (Leicester) [23]	0.955	0.017	0.028
Ethnic minorities (Leicester) [23]	0.931	0.024	0.045
White (Surrey) [1,5]	0.909	0.074	0.017
Ethnic minorities (Surrey) [1,5]	0.919	0.024 *	0.057

^#^ Proportion with normal glucose tolerance = 1–Proportion with high risk of diabetes–proportion with type 2 diabetes. * Data unavailable. Assumed similar to Leicester [23].

**Table 2:** Demographics and test results for individuals within two pilot sites.

Demographic	Leicester (n = 172) No. (%)	Surrey (n = 164) No. (%)
Female	99 (57.2)	110 (67.1)
White European	98 (57)	106 (64.6)
Age group		
<50	93 (54.1)	109 (66.5)
50–59	27 (15.7)	25 (15.2)
60–69	22 (12.8)	21 (12.8)
70+	30 (17.4)	9 (5.5)
**CRF Test Results**	**(n = 172)**	**(n = 164)**
Low risk (<16)	108 (62.8)	124 (76.6)
Medium to Hhgh risk (16+)	64 (37.1)	40 (24.4)
**Available HbA1c Test Results**	(n = 33) *	(n = 31) *
Suspected diabetes ≥6.5% (48mmol/mol)	5	3
High risk of-diabetes 6.1% (43mmol/mol) to 6.4% (47mmol/mol)	5	5
Unlikely to have diabetes ≤6% (42mmol/mol)	23	23
**Predicted HbA1c Test Results**		
Suspected diabetes ≥6.5% (48mmol/mol)	9 ^#^ (5.2)	4 ^#^ (2.4)
High risk of diabetes 6.1% (43mmol/mol) to 6.4% (47mmol/mol)	9 ^#^ (5.2)	6 ^#^ (3.9)
Unlikely to have diabetes ≤6% (42mmol/mol)	45 ^#^ (26.2)	30 ^#^ (18.1)

* Number of results available for analysis which is less than the number of patients actually screened; # Predicted results assuming data available for all patients screened at risk with customer service record forms (CRFs) available i.e. n = 64 for Leicester and n = 40 for Surrey.

**Table 3:** Predictive Probabilities for Screening Outcomes.

	White Leicester	White Surrey	Ethnic Minority Leicester	Ethnic Minority Surrey
	Numbers (Proportions)			
**CRF Questionnaire**				
Total numbers	97	134	75	30
**Screen High/Moderate Risk**	**53.9 (0.556)**	**76.4 (0.57)**	**41.9 (0.558)**	**16.8 (0.558)**
True type 2 diabetes (T2D)	0.33 (0.006)	0.28 (0.004)	0.41 (0.01)	0.21 (0.013)
True high risk of diabetes	1.34 (0.025)	8.03 (0.105)	1.46 (0.035)	0.58 (0.035)
False positives	52.2 (0.969)	68.1 (0.891)	40 (0.955)	16 (0.953)
**Screen Low Risk**	**43.1 (0.444)**	**57.6 (0.43)**	**33.1 (0.442)**	**13.2 (0.442)**
True Negatives	42.7 (0.991)	55.7 (0.966)	32.7 (0.987)	13.1 (0.986)
False Negatives – True T2D	0.08 (0.002)	0.07 (0.001)	0.1 (0.003)	0.05 (0.004)
False Negatives – High risk of diabetes	0.31 (0.007)	1.88 (0.033)	0.34 (0.01)	0.14 (0.01)
Assume all high/moderate risk from CRF receive HbA1c				
Numbers	53.9 (0.556)	76.4 (0.57)	41.9 (0.558)	16.8 (0.558)
HbA1c blood test +ve	16.1 (0.298)	23.9 (0.313)	12.7 (0.303)	5.1 (0.304)
**Screen High Risk**	**4.46 (0.278)**	**5.68 (0.238)**	**3.55 (0.281)**	**1.46 (0.286)**
TrueT2D	0.29 (0.02)	0.24 (0.01)	0.36 (0.03)	0.18 (0.04)
False Positives	4.18 (0.26)	5.44 (0.23)	3.2 (0.25)	1.28 (0.25)
**Screen Moderate Risk**	**11.6 (0.722)**	**18.2 (0.762)**	**9.1 (0.719)**	**3.6 (0.714)**
True high risk of-diabetes	0.65 (0.041)	3.94 (0.71)	0.71 (0.29)	0.29 (0)
False Positives	11 (0.682)	14.3 (8.4)	8.4 (3.4)	3.4 (0)

**Table 4:** Unit Costs per Individual.

Cost Item	Total Cost	Cost Per Test	Assumption
Costs of providing CRF screening			
Training (annual equivalent cost)	£55	£0.74	73 people screened per year in each practice
Total marketing (including leaflets, posters, hanging cards)	£155	£2.11	
Printing: service/clinical/customer feedback forms	£26	£0.35	
Healthcare assistant (HCA)	£235	£3.23	
Total cost of CRF	£471	£6.44	
Cost of HbA1c test			40.9 out of 73 individuals will receive HbA1c testing
HbA1c Analyser (annual equivalent cost)	£328	£8.02	
Internal quality control, annual (6 samples)	£72	£1.76	
External quality assurance, annual (6 samples)	£216	£5.29	
Ecoloc bins collection annual fee	£67	£1.64	
Pharmacist	£748	£18.29	
Consumables	£190	£4.64	
Total for HbA1c	£1621	£39.64	
Average cost per person screened in pharmacy		£28.65	

**Table 5:** Estimated cost of identifying true type 2 diabetes *.

Costs 2017 £ Sterling	Leicester	Surrey	Total
Costs of CRF screening	£1109	£1057	£2166
Costs of HbA1c test	£3795	£3691	£7486
Total costs of Screening	£4903	£4748	£9651
Number appropriately referred	0.642	0.420	1.062
Cost per appropriately referred individual	£7638	£11,297	£9086
Cost per appropriately referred individual assuming 60% attend medical practice	£12,730	£18,828	£15,142

* Data provided are subject to rounding errors within the underpinning data and are therefore approximations.
